# Astaxanthin Protects OTA-Induced Lung Injury in Mice through the Nrf2/NF-κB Pathway

**DOI:** 10.3390/toxins11090540

**Published:** 2019-09-17

**Authors:** Weixiang Xu, Mingyang Wang, Gengyuan Cui, Lin Li, Danyang Jiao, Beibei Yao, Ketao Xu, Yueli Chen, Miao Long, Shuhua Yang, Jianbin He

**Affiliations:** 1 Key Laboratory of Zoonosis of Liaoning Province, College of Animal Science & Veterinary Medicine, Shenyang Agricultural University, Shenyang 110866, China; 2018240355@stu.syau.edu.cn (W.X.); m13940546231@163.com (M.W.); 2018240341@stu.syau.edu.cn (G.C.); 2017200134@stu.syau.edu.cn (L.L.); 2018240365@stu.syau.edu.cn (D.J.); 2018240335@stu.syau.edu.cn (B.Y.); 2018240362@stu.syau.edu.cn (K.X.); 2018220508@stu.syau.edu.cn (Y.C.); 2 Department of Epidemic Control Fushun modern agriculture and poverty alleviation and development promotion center, Fushun 113006, China

**Keywords:** ochratoxin, astaxanthin, oxidative stress, inflammation, Nrf2 pathway, NF-κB pathway, mouse, lung

## Abstract

The aim of this research was to evaluate the potential protective mechanism of astaxanthin (ASTA) against oxidative damage and inflammation caused by ochratoxin (OTA) in mouse lung. We divided mice into a control group (CG), an OTA group (PG), an astaxanthin group (AG), and an OTA+ASTA group (JG). Oxidative indices (malondialdehyde (MDA), total superoxide dismutase (T-SOD), and reduced glutathione (GSH)) and inflammatory markers (interleukin 1β (IL-1β), interleukin 6 (IL-6), and tumor necrosis factor α (TNF-α)) were assayed in the lung, and the lung-weight-to-body-weight ratio was calculated. Apoptosis was detected in pathological sections by the TdT-mediated dUTP nick-end labeling (TUNEL) assay. Oxidative damage and inflammation were detected in the lung of mice after exposure to OTA. Besides, Nrf2- and NF-κB-pathway-associated proteins were detected by Western blot. In contrast with OTA, ASTA significantly raised the expression of Nrf2, HO-1, and MnSOD, while the expression of other proteins (Keap1, TLR4, and NF-κB) was significantly decreased. These results indicate that ASTA exerted protective effects against OTA-induced oxidative damage and inflammation in the lung by regulating the Nrf2 and NF-κB pathways.

## 1. Introduction

Ochratoxin (OTA) is a mycotoxin produced by the secondary metabolism of two genera of fungi, i.e., *Penicillium* and *Aspergillus*. OTA is a white crystalline compound, which is insoluble in water and can be dissolved in sodium bicarbonate organic solutions, especially in polar organic solvents [1]. Its contamination is always clearly visible, appearing as mold on food and feed, commonly in cereal grains [2]. Several animal studies have shown that OTA may be carcinogenic to humans, and in fact it has been classified as a human carcinogen in Group 2B by the International Agency for Research on Cancer (IARC) [3]. Studies have indicated that OTA can damage tissues and organs after entering the human or animal body, causing kidney damage, liver damage, embryo damage, nerve damage, immune damage, teratogenesis, and carcinogenesis [4,5,6,7]. Studies by Pinelli et al. demonstrated that eight DNA adducts were detected 4 hours after exposure to human epithelial lung cells with 10 μM OTA [8], which indicates the genotoxicity of OTA to bronchial epithelial cells. This experiment is important because acute renal failure is observed after inhalation of mycotoxins [9]. Through inhalation, toxins or their metabolites can enter the blood directly, instead of re-entering the blood through the liver or kidney circulation. In vivo experiments by Zheng et al. showed that after continuous oral administration of OTA for 20 days, OTA concentration in the lungs is the highest, followed by the liver, heart, and kidney [10]. Studies have shown that OTA not only produces oxidative stress in the kidney by activating the NRF2 signaling pathway [11] but also is a potent inducer of apoptosis [12]. In addition, OTA can also infect the lungs by inhalation [13]. Okutan et al. showed that OTA caused damage to rat hearts and lungs [14]. The main mechanisms of OTA toxicity are inhibition of protein synthesis, oxidative stress, DNA molecular damage, and damage to calcium homeostasis. In 1984, Creppy et al. found that OTA inhibited the expression of aminoacyl-tRNA synthetase, valyl-tRNA synthetase, and phenylalanine-tRNA synthetase, thus reducing protein synthesis [15]. Studies by Khan et al. showed that OTA can inhibit the ATP-dependent calcium uptake rate by 42–45% [16]. According to a study by Jing Liu et al., OTA stimulates the body to produce oxidative stress, and OTA-induced damage to DNA molecules may be related to OTA carcinogenesis. [17]. However, the molecular mechanism of OTA-induced lung injury has not been reported yet. 

Astaxanthin (ASTA) belongs to the carotenoid family, a new generation of powerful antioxidants that have received much attention in recent years. This non-toxic natural carotenoid is found in yeast and marine organisms such as algae, fish, and shrimps [18,19]. ASTA has a strong antioxidant capacity due to its chemical structure which is characterized by the 3, 3′ positions of the β-ionone ring with hydroxyl group (-OH) on either end of the molecule [20]. Naguib and Fukuzawa showed that ASTA had a stronger antioxidant capacity than other beta carotenoids or vitamin E [21,22]. Its antioxidant capacity was more than 10 times that of other beta carotenoids and 100–500 times that of vitamin E [23]. ASTA not only has a protective effect on oxidative stress [24] but also has a protective effect against inflammation and apoptosis [25,26,27,28]. Recent studies reported that ASTA had a protecting effect against mycotoxins [29,30,31], and Peteri et al. reported that OTA can be degraded and adsorbed by yeast capable of producing astaxanthin [32]. However, neither the detoxification effect of ASTA on OTA nor its mechanism of protection has been reported in vivo.

Our research validated the protective effect of ASTA on lung injury induced by OTA in mice by constructing an OTA mice model and elucidated the mechanism of protection by molecular experiments. The results provide a molecular basis for the toxicological mechanism of OTA in the lung and the protective effect of ASTA on OTA toxicity.

## 2. Results

### 2.1. Physiological Index

During feeding, all mice had normal appetite, behavior, fur gloss, and urine output. During intragastric administration of OTA, ASTA, OTA+ASTA, or olive oil (control), mice in the control group (CG) and the ASTA group (AG) had normal appetite, normal behavior, normal urine output, and smooth back hair. In contrast, the back hair of mice in the OTA group (PG) was rough, they showed poor mental state, and their urine output was significantly increased. The mice in the OTA+ASTA group (JG) were a healthier state than those in the PG.

### 2.2. Mice Lung Organ Ratios

**Figure 1:**
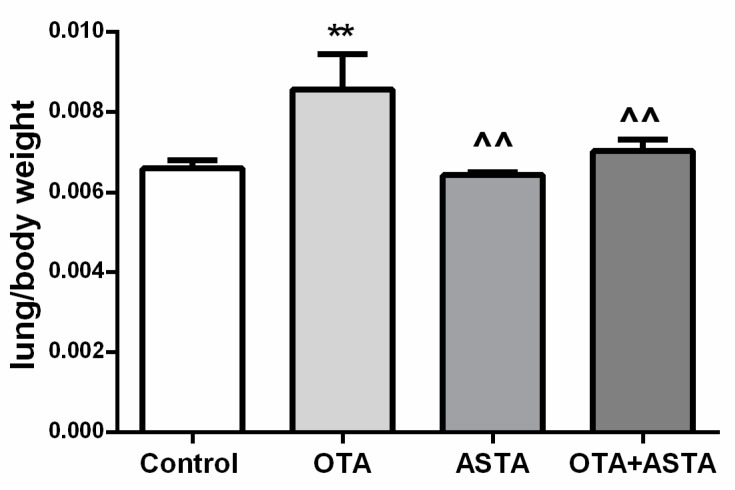
Changes in lung-weight-to-body-weight ratios in mice. OTA: ochratoxin, ASTA: astaxanthin; ** indicates a significant difference compared to the control group (CG) (*p* < 0.01); ^^ indicates a significant difference compared to the OTA+ASTA group (PG) (*p* < 0.01).

As shown in Figure 1,after 28 days of in vivo experiments, the lung-weight-to-body-weight ratio of the PG was much higher than that of the CG (*p* < 0.01). There was no significant difference in organ ratio between AG and JG group compared with CG group (*p* > 0.05). The lung-weight-to-body-weight ratios of the AG and JG were much smaller than that of the PG (*p* < 0.01).

### 2.3. Pathological Changes in Lung Organ

**Figure 2:**
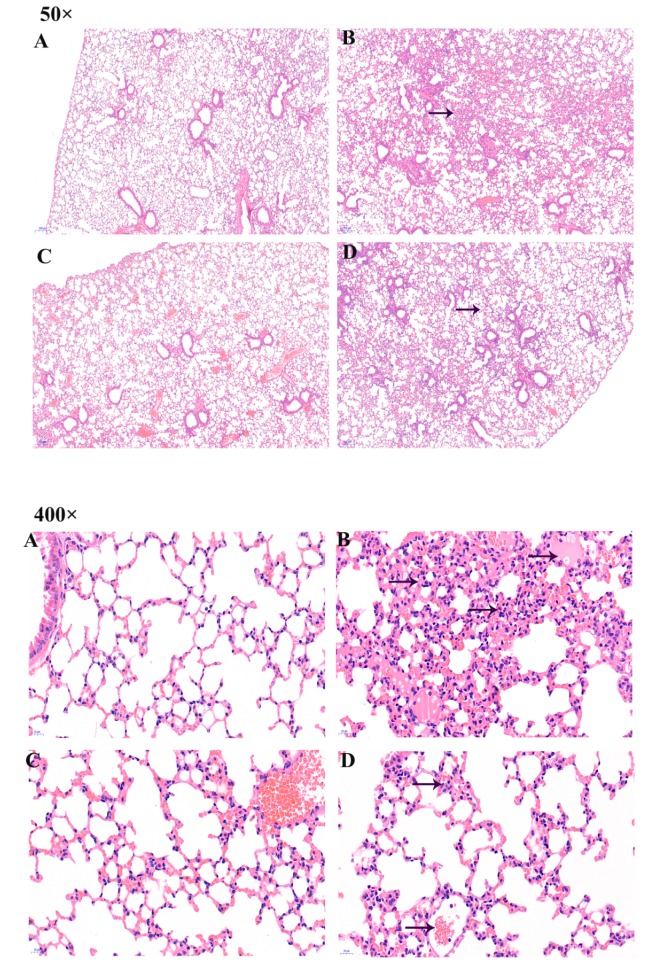

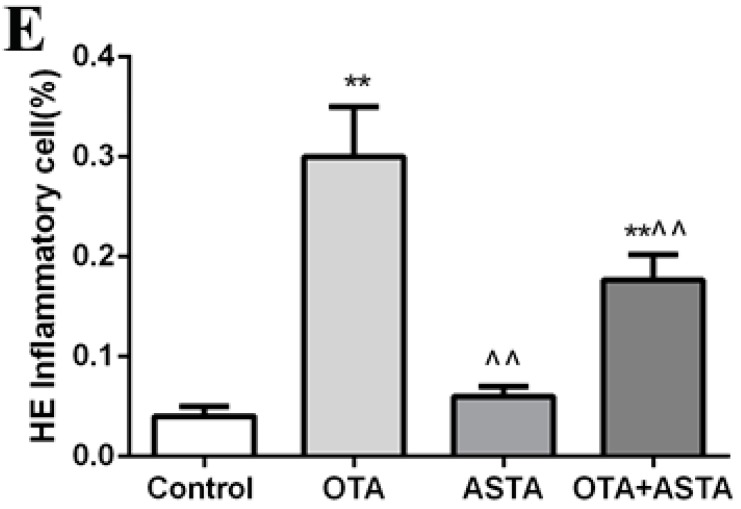
Pathological changes in the lungs were detected by tissue section H&E staining. Images were taken at magnifications of 50× and 400×. (**A**) CG, (**B**) OTA (5 mg/kg body weight) group, (**C**) ASTA (100 mg/kg body weight) group, and (**D**) ASTA + OTA (5 mg/kg body weight) group. The arrow → indicates pathological damage in the lungs, such as pulmonary interstitial widening, hyperemia, and alveolar rupture. (**E**) Quantitative assessment of inflammatory cells. "**" indicates a significant difference with respect to CG (*p* < 0.01); “^^” indicates a significant difference with respect to PG (*p* < 0.01).

Hematoxylin-eosin staining (H&E);staining was used to observe lung histological changes. In CG mice, the alveolar walls of the lungs were normal, and the alveolar septum was not infiltrated. No inflammation, congestion, bleeding, or exudate were observed (Figure 2A). In contrast, the lungs of mice in the PG showed hyperemia, hemorrhage, exudation, alveolar rupture, pulmonary interstitial broadening, and extensive inflammatory cell infiltration and aggregation of foam macrophages (Figure 2B). There was no significant change in AG mice compared to CG mice (Figure 2C). Compared to CG mice, the lungs of JG mice presented some damage, but it was less diffuse than that seen in PG mice (Figure 2D).

As can be seen from the histogram, the inflammatory cells in PG lungs covered 30% of the total lung area, which could lead to necrosis of the lungs (Figure 2E). The inflammatory cells in CG and AG lungs were rare, while in JG lungs they were more than in CG lungs, but less than in PG lungs, indicating that ASTA had a certain therapeutic effect in OTA-treated mice.

### 2.4. Analysis of Apoptosis by TUNEL in Mouse Lung

**Figure 3:**
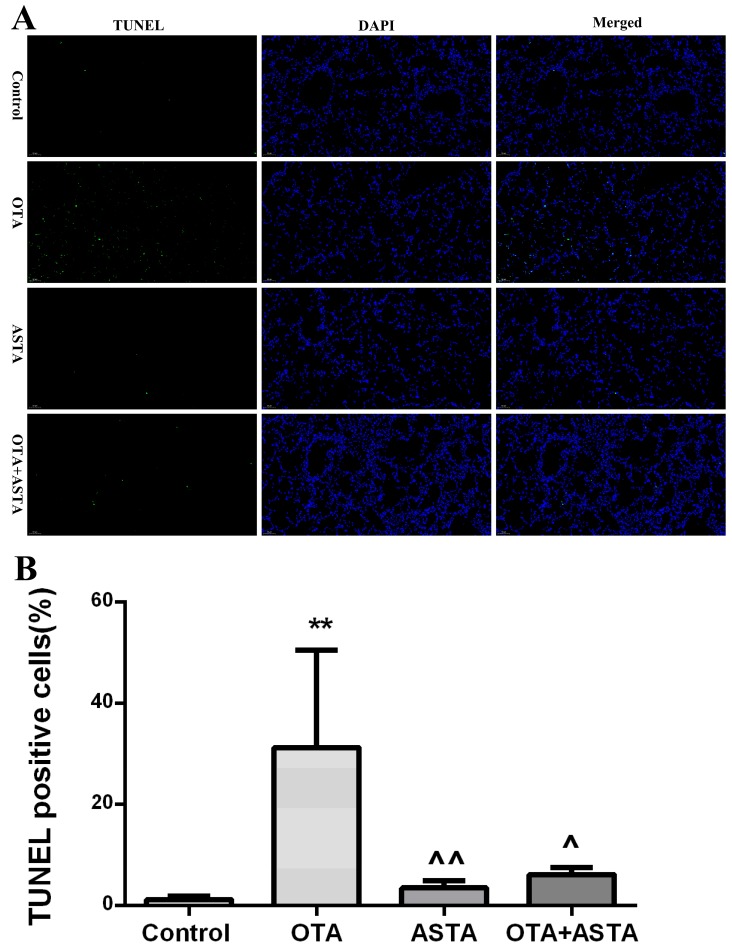
(A) TUNEL staining. Apoptosis was analyzed in four groups using the TUNEL assay. Green fluorescence indicates TUNEL-positive cells in the microscopic field. DAPI was used for nuclear staining (magnification 200×). (B) TUNEL-positive cells. ** indicates a significant difference with respect to CG (*p* < 0.01); ^ indicates a significant difference (*p* < 0.05) with respect to PG; ^^ indicates a significant difference (*p* < 0.01) with respect to PG (magnification 200×).

As shown in Figure 3A, green fluorescence represents TUNEL positive cells. It can be seen that the green fluorescence in PG lungs was particularly high, which indicates that there was a large number of apoptotic cells in the lungs of OTA mice. In contrast, the green fluorescence in CG and AG mice was very limited. Although there was apoptosis in the lungs of these groups, it was rarely detectable. The amount of green fluorescence in JG lungs was slightly higher than that in CG lungs, but it was lower than the amount of green fluorescence in OTA lungs, indicating that ASTA had a certain inhibitory effect on apoptosis induced by OTA. 

As shown in Figure 3B below, the percentage of TUNEL-positive cells was much higher in PG than in CG (*p* < 0.01), was similar in AG and in CG (*p* > 0.05), and was slightly higher in JG than in CG (*p* > 0.05); also, it was much lower in ASTA than that in OTA (*p* < 0.05) and in JG than in PG (*p* < 0.05).

### 2.5. Changes of the Oxidation Index in the Lungs

**Figure 4:**
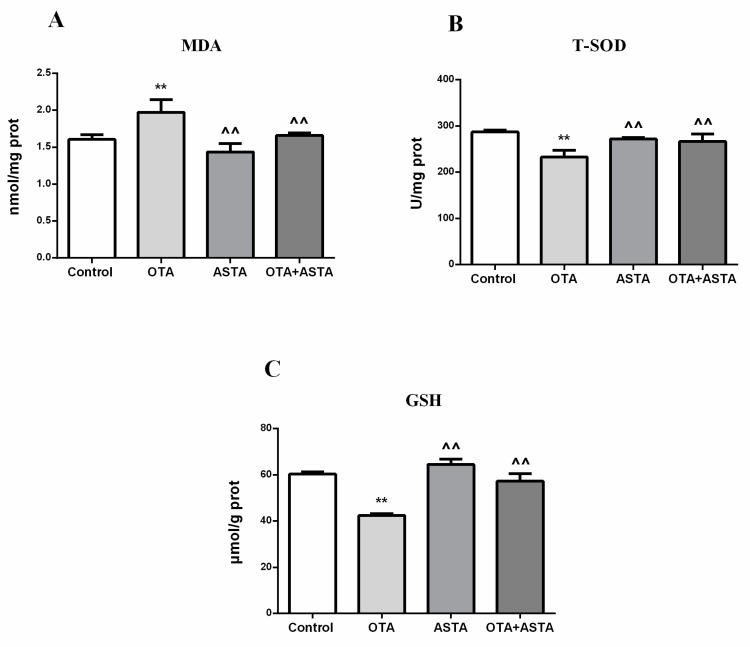
Content of various oxidation indicators in the lungs of mice (**A**) Malondialdehyde (MDA) concentration, (**B**) total superoxide dismutase (T-SOD) concentration, and (**C**) reduced glutathione (GSH) concentration. ** indicates a significant difference with respect to CG (*p* < 0.01); ^^ indicates a significant difference with respect to PG (*p* < 0.01).

We determined whether OTA and ASTA caused oxidative stress by measuring the oxidation index in the lungs of each group, on the basis of their content of malondialdehyde, total superoxide dismutase, and reduced glutathione. As shown in Figure 4, the amount of malondialdehyde in PG lungs was much higher than in CG lungs, while those of total superoxide dismutase and reduced glutathione were much lower than in CG lungs. The content of malondialdehyde, total superoxide dismutase, and reduced glutathione was basically unchanged in AG lungs compared with CG lungs and did not change significantly in JG lungs compared with CG lungs.

The malondialdehyde content in AG lungs was much lower than in PG lungs, while those of total superoxide dismutase and reduced glutathione were much higher than in PG lungs. The malondialdehyde amount in JG lungs was much lower than in PG lungs, while the total superoxide dismutase and reduced glutathione contents were much higher than in PG lungs.

### 2.6. Changes in Inflammation Markers in the Lungs

**Figure 5:**
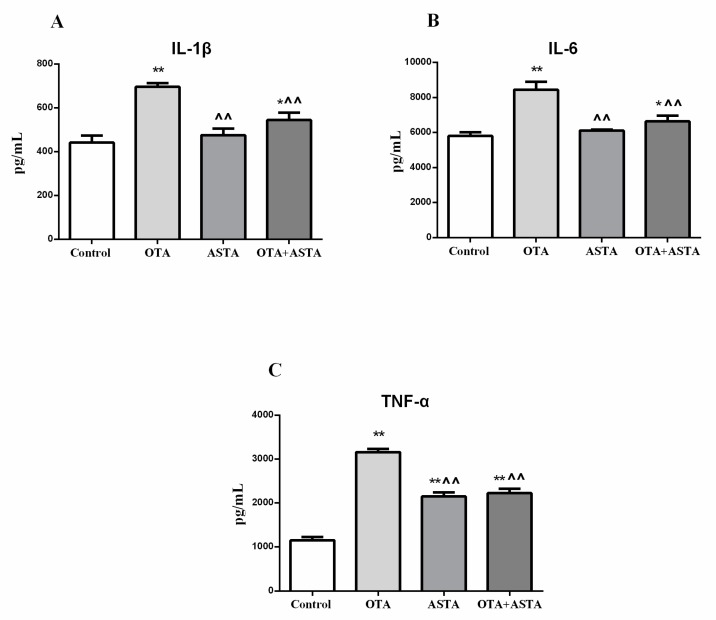
Content of various inflammatory markers in the lungs. (**A**) Interleukin-1βconcentration, (**B**) interleukin-6 concentration, and (**C**) tumor necrosis factor α concentration. * indicates a significant difference (*p* < 0.05) compared to CG, and ** indicates a very significant difference (*p* < 0.01) compared to CG; ^^ indicates a very significant difference (*p* < 0.01) compared to PG.

We determined whether OTA and ASTA caused an inflammatory response in mice lungs by measuring inflammation markers, such as interleukin-1β, interleukin-6, and tumor necrosis factor α. As shown in Figure 5, the amounts of interleukin-1β, interleukin-6, and tumor necrosis factor α in PG lungs were much higher than those in CG lungs. The tumor necrosis factor α amounts in AG lungs were much higher than those in CG lungs, while interleukin-1β and interleukin-6 amounts in AG lungs were basically unchanged compared with CG lungs. The interleukin-1β, interleukin-6, and tumor necrosis factor α amounts in JG lungs were increased compared with those in CG lungs.

The values of interleukin-1β, interleukin-6, and tumor necrosis factor α in AG and JG lungs were much lower than those in PG lungs.

### 2.7. Changes in the Expression of Nrf2, Keap1, HO-1, and MnSOD in Mice Lungs

**Figure 6:**
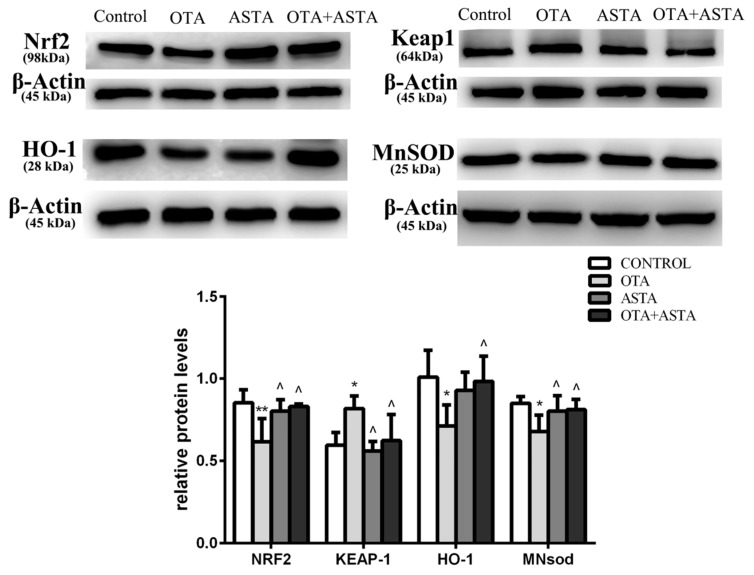
Effects of OTA and ASTA on the expression of Nrf2, Keap1, HO-1, and MnSOD proteins in the lungs. * indicates a significant difference (*p* < 0.05) compared to CG, and ** indicates a very significant difference (*p* < 0.01) compared to CG; ^ indicates a significant difference (*p* < 0.05) compared to PG.

As shown in Figure 6, the expression of Nrf2 in PG lungs was much lower than that in CG lungs, the expression of HO-1 and MnSOD was lower than that in CG lungs, and the expression of Keap1 was higher than that in CG lungs. The expression levels of Nrf2, HO-1, MnSOD, and Keap1 in AG lungs were similar to those in CG lungs. The expression levels of NRF2, HO-1, MnSOD, and Keap1 in JG lungs were similar to those in CG lungs.

The expression of Nrf2, HO-1, and MnSOD in AG lungs was higher than in PG lungs, while the expression of Keap1 was lower than in PG lungs. The expression of Nrf2, HO-1, and MnSOD in JG lungs was higher than in PG lungs, while the expression of Keap1 was lower than in PG lungs. 

### 2.8. Changes in the Expression of TLR4, MyD88, and NF-κB in Mice Lungs

**Figure 7:**
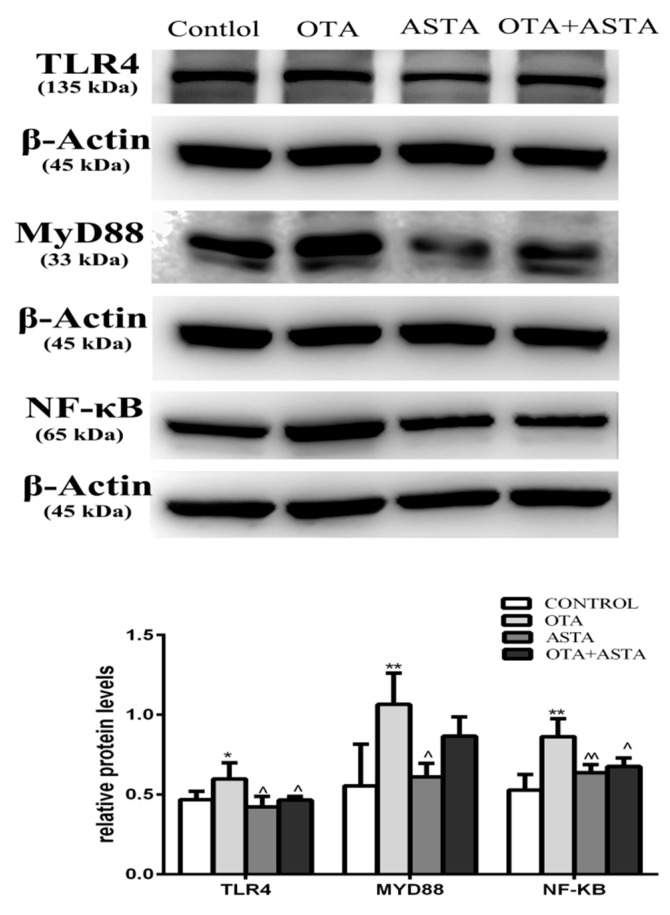
Effects of OTA and ASTA on the expression of TLR4, MyD88, and NF-κB in the lungs. * indicates a significant difference (*p* < 0.05) compared to CG. and ** indicates a very significant difference (*p* < 0.01) compared to CG; ^ indicates a significant difference (*p* < 0.05) compared to PG, and ^^ indicates a very significant difference (*p* < 0.01) compared to PG.

As shown in Figure 7, the expression of TLR4 in PG lungs was much higher than that in CG lungs, and the expression of MyD88 and NF-κB was higher than in CG lungs. The expression of TLR4, MyD88, and NF-κB in AG and JG lungs was similar to that in CG lungs. 

The expression of TLR4 and MyD88 in AG lungs was lower than that in PG lungs, and the expression of NF-κB was much lower than that in PG lungs. The expression of TLR4 and NF-κB in JG lungs was lower than that in PG lungs, and the expression of MyD88 was similar to that in PG lungs.

## 3. Discussion

In our research, we explored the protective effects of ASTA on OTA-induced lung injury. Previous studies have shown that OTA reduced sperm motility [33], induced hepatotoxicity and nephrotoxicity, and produced oxidative stress, inflammation, and apoptosis in various cells and tissues [34,35,36]. Okutan et al. showed that OTA had toxic effects resulting in damage to the heart and lungs [14], while ASTA was shown to protect against oxidative stress, inflammation, and apoptosis in other studies. We demonstrated the detoxification activity of ASTA on OTA by showing its effects on protein expression, oxidation and inflammation markers, and apoptosis.

Janette Hope et al. found that the urine volume of animals poisoned by OTA was increased compared to that of healthy controls [37]. In our study, the physiological indicators of PG and JG mice confirmed this phenomenon. We performed a daily weight record of the mice. We found that the lung-weight-to-body-weight ratio in PG mice was increased compared to that of mice in the other groups because the weight loss of the mice in the PG was particularly significant. This suggested that the mice in the PG had been poisoned, and the results from H&E and TUNEL staining showed that ASTA had a significant detoxification effect in OTA-treated mice.

The activity of malondialdehyde, total superoxide dismutase, and reduced glutathione can be indicative of oxidative damage. Previous studies have shown that OTA can produce oxidative stress in the body. Under normal conditions, the levels of oxidants and antioxidants in the system are balanced [38]. In our study, we found that OTA induced oxidative damage in the lungs, which is consistent with previous reports. In addition, oxidative damage to the lungs by OTA was significantly altered by ASTA. The results showed that ASTA had a protective effect on OTA-induced lung oxidative damage.

Nrf2 is a regulatory protein largely distributed in various organs of the body. It is the main regulator of cellular redox reactions [39]. Under normal physiological conditions, Nrf2 binds to Kelch-like ECH-related protein 1 (Keap1), which exists in the cytoplasm in its inactive state, and rapidly degrades under the action of the ubiquitin-proteasome pathway, which maintains a low transcription level of Nrf2 under physiological conditions [40]. When toxic substances enter the human body, Nrf2 is uncoupled from Keap1, and activated Nrf2 is transported into the nucleus. The translocated Nrf2 binds to the antioxidant response element (ARE) and induces the expression of detoxification and antioxidant enzymes, thereby exerting antioxidant activity [8]. Numerous studies have shown that Nrf 2 exerts broad protection against a variety of diseases caused or exacerbated by oxidative stress [41,42,43,44]. Nrf2 controls the expression of various antioxidant enzymes, such as HO-1, MnSOD, etc. [45], thereby inhibiting cell damage caused by oxidative stress. Only Keap1 expression was elevated in PG lungs compared to CG lungs, while Nrf2, HO-1, and MnSOD expression was decreased, which may have been due to the long-term chronic OTA challenge. Antioxidant reactions consume a large amount of antioxidant enzymes. When JG lungs were compared with PG and CG lungs, the toxicity of in JG appeared relieved, which proved that ASTA was able to protect against the toxic effects of OTA.

Previous studies have shown that OTA mediated immunotoxicity of the TLR4/MyD88 signaling pathway by inducing an increase in reactive oxygen species in porcine alveolar macrophages [46]. According to our results, when pretreated with ASTA, the lung interstitial broadening seen after OTA-induced lung injury in mice was significantly attenuated, and inflammation marker (interleukin-1β, interleukin-6, and tumor necrosis factor α) levels were reduced. This suggests that ASTA played a role in relieving lung inflammation caused by OTA. The tumor necrosis factor α levels in AG lungs were significantly different from those in CG. Although previous studies do not agree with these results, we think ASTA may cause a certain inflammatory response in the lungs of mice. TLRs are one of the most important pattern recognition receptors and play a key role in the induction of inflammatory responses and the production of inflammatory mediators [47]. While TLR4 is a key mediator of the pro-inflammatory response, expression and activation of the TLR 4/MyD 88/NF-κB pathway promote the production and release of inflammatory cytokines and biological mediators [48]. NF-κB is an important regulatory hub that regulates ROS, inflammatory mediators, and pro-inflammatory factors. [49]. Under normal physiological conditions, NF-κB remains in the cytoplasm along with IκB. IκB kinase (IKK) is activated first when the receptor protein is stimulated. IKK phosphorylates a serine residue of the IκB subunit regulatory site in the NF–κB·IκB complex in the cell, so that the IκB subunit is ubiquitinated and further degraded by proteases, thereby releasing the NF-κB dimer. Free NF-κB enters the nucleus and binds to genes with NF-κB binding sites to initiate transcriptional processes [50]. Previous studies have shown that OTA caused inflammation in mice spleens through NF-κB [2]. In our study, levels of TLR4, MyD88, and NF-κB were increased in PG lungs compared to CG, indicating inflammation. However, TLR4, MyD88 and NF-κB were reduced in JG lung compared to CG, indicating a reduction in lung toxicity in JG. And proved that ASTA can resist the toxic effects of OTA.

## 4. Conclusions

In summary, ASTA reduced lung oxidative damage in mice induced by OTA exposure through the Nrf2 signaling pathway. ASTA also reduced lung inflammation in mice induced by OTA exposure through the NF-κB signaling pathway. This is the first study showing ASTA-mediated protection of OTA-induced oxidative stress and inflammation through the Nrf2 and NF-κB pathways.

## 5. Materials and Methods 

### 5.1. Animals

Eighty C57 mice (20 ± 2 g, six-week old) were provided by Shandong Jinan Pengyue Experimental Animal Breeding Co., Ltd. After transport, the mice were acclimated to the environment for three weeks prior to the experiments. They were housed at room temperature (22–24 °C) and subjected to a 12 h light/dark cycle at 40–60% relative humidity. The lights were turned on at 6:30 every morning, and feeding (Changsheng Biotechnology Co., Ltd.) was stopped; then the lights were turned off at 6:30 pm, and feeding was started. The litter was changed every two days (Liaoning Changsheng Biotechnology Co., Ltd., Benxi, China). Water was uninterrupted. The experimental procedures were approved by the Ethics Committee for Laboratory Animal Care (Animal Ethics Procedures and Guidelines of the China) for use by Shenyang Agricultural University, China (Permit No. 264 SYXK<Liao>2011-0001, 20 October 2011).

### 5.2. Experimental Design and Treatment

OTA was purchased from LKT Labs (St. Paul, MN, USA) and dissolved in 0.1 mol/L sodium bicarbonate (NaHCO3). ASTA was from *Haematococcus pluvialis*, was purchased from Solarbio company (Solarbio, Beijing, China), and dissolved in 1 kg/L olive oil.

The mice were divided into the CG, PG, AG, and JG (20 mice per group). The mice received test substances by gavage for 27 days, each day for seven days, then were rested for two days, for a total of four weeks. To mice in the CG, 0.1 ml of olive oil was administered intragastrically daily, then 0.1 ml of NaHCO3 was given two hours later. In the PG, gavage administration was carried out according to the study of Hibi et al. [51]. OTA (0.1 ml, 5 mg/kg body weight) was administered two hours after the first gavage of 0.1 ml olive oil. ASTA was given according to the study by Zhao et al. [52]. ASTA (0.1 ml, 100 mg/kg body weight) was administered first, then 0.1 ml of NaHCO3 solution was administered two hours later. To mice in the JG, 0.1 ml of ASTA (100 mg/kg body weight) was given by gavage, and 0.1 ml OTA (5 mg/kg body weight) was administered two hours later. 

### 5.3. Pulmonary Organ Indices

The body weights of all mice were measured 24 h after the last treatment. Then, the mice were anesthetized, and the lungs were removed. The organ coefficient was calculated as the total weight of the organ wet weight (g)/mouse body weight (g).

### 5.4. Hematoxylin and Eosin (H&E) Staining

The lungs of three mice per group were excised and fixed in 10% neutral formalin, then dehydrated in an ethanol series, immersed in wax, and subsequently embedded in paraffin. The tissues were cut into 4 μm-thick sections, stained with hematoxylin and eosin (H&E) (Servicebio, Wuhan, China), and examined by optical microscopy using a Leica DM750 microscope (Leica, Beijing, China) to assess histopathological damage.

### 5.5. TUNEL Apoptosis Analysis

TUNEL analysis was performed using commercial kits according to the manufacturer’s instructions. The specific procedure was as follows: paraffin-embedded mice lung sections were fixed in xylene for 15 min, washed twice with absolute ethanol for 3 minutes, then washed with phosphate-buffered saline (PBS) for 5 minutes. After the sections were fixed, they were incubated with 100 μL (20 μg/mL) of proteinase K solution for 10 minutes. Reaction buffer (10 μL of 5× buffer), 38 μL of double-distilled H2O (ddH2O), 1 μL of fluorescein-isothiocyanate (FITC)-labeled dUTP, and 1 μL of terminal deoxynucleotidyl transferase enzyme solution were mixed at room temperature and added to the section. The samples were then stained with DAPI (4’,6-diamidino-2-phenylindole) for 8 minutes. The sections were washed and photographed under a fluorescence microscope.

### 5.6. Oxidation Assays in Mice Lungs 

The lungs were homogenized using a tissue homogenizer and centrifuged at 2500 rpm for 10 minutes, and the supernatants were collected. Test methods for various indicators followed the manufacturer’s instructions (Nanjing Jiancheng Bioengineering Institute, China). For example, malondialdehyde was tested by the thiobarbituric acid method (TBA)method, total superoxide dismutase was tested by the hydroxylamine method, and reduced glutathione was tested by a microplate method.

### 5.7. Inflammation Indices in Mice Lungs 

The lungs were homogenized using a tissue homogenizer, a part of the lung suspensions was centrifuged at 3000 rpm for 20 minutes, and thes supernatants were collected. Test methods for each indicator followed the manufacturer’s instructions (Jiangsu Meimian Industrial Co., Ltd, Jiangsu, China). 

### 5.8. Western Blot Analysis

The lungs (0.1 g) were mixed with 0.99 mL of RIPA lysate (Solarbio, Beijing, China) and 0.01 mL of protease inhibitor PMSF (Solarbio, Beijing, China) into a tissue homogenizer to obtain homogenates. After standing for 30 minutes, the tissue supernatant was centrifuged at 120,000 g at 4 °C for 25 min, and then the intermediate liquid was transferred to a new enzyme-free centrifuge tube using a pipette. Protein concentration was quantified using a BCA kit (Solarbio, Beijing, China).

After denaturation, the proteins (15 μg) were transferred to a gel plate by electrophoresis and then to an Immobilon-membrane (0.45 μM) by electroporation. The membrane was soaked in 5% skim milk (Solarbio, Beijing, China) and then blocked in a shaker at room temperature for 2 hours. Then, the membrane was incubated with primary antibody solutions (CST, Boston, MA, USA), i.e., anti-Nrf2 (1:1000) diluted with milk powder and PBST, anti-Keap1 (1:1000), anti-HO-1 (1:1000), anti-MnSOD (1: 1000), anti-TLR4 (1:1000), anti-MyD88 (1:1000), anti-NF-κB (1:1000), and anti- β-actin (1:1000) overnight at 4 °C. The next day, after washing 5 times with PBST, the membrane diluted in PBST was incubated with HRP-conjugated secondary antibodies (1:3000) (CST, USA) for one hour at room temperature. Finally, the membrane was exposed after washing 3 times with PBST using an ECL chemiluminescence solution (Solarbio, Beijing, China). The DNR bioimaging system (Ncmbio, suzhou, China) was used to visualize the relative intensity of the bands. The relative density of the proteins of interest was calculated using ImageJ software. The expression level of the target proteins was determined by calculating the ratio between the intensity of the target protein band and that of β-actin band, so that the protein expression levels for the different groups of samples were comparable.

### 5.9. Statistical Analysis

Statistical analysis was performed using SPSS 22.0 software (IBM Corporation, Armonk, New York, NY, USA), and multiple comparisons were performed by analysis of variance (ANOVA) and LSD methods. Data are expressed as mean ± standard deviation of at least 3 independent experiments; *p* < 0.05, indicates significant difference; *p* < 0.01, indicates extremely significant differences.
